# Anxiety, Attachment Styles and Life Satisfaction in the Polish LGBTQ+ Community

**DOI:** 10.3390/ijerph20146392

**Published:** 2023-07-19

**Authors:** Zofia Kardasz, Rafał Gerymski, Arkadiusz Parker

**Affiliations:** 1 Department of Social Clinical Psychology, Institute of Psychology, Opole University, 45-040 Opole, Poland; zofia.kardasz@uni.opole.pl; 2 Department of Health Psychology and Quality of Life, Institute of Psychology, Opole University, 45-040 Opole, Poland; 3 PROMENADA Foundation, 45-753 Opole, Poland

**Keywords:** anxiety, attachment styles, life satisfaction, sexual minority, LGBT

## Abstract

Poland is one of the most discriminatory places in Europe. The political situation, legal policies, and society’s attitude towards people from the LGBTQ+ community in Poland clearly indicate the need to study the mental health and well-being of this group of individuals. Based on Meyer’s minority stress theory, Bowlby’s attachment theory, the Ainsworth attachment framework, Diener’s subjective well-being model, and provided empirical evidence, this study examined the significance of attachment styles and anxiety as predictors of life satisfaction among the Polish LGBTQ+ community. It also explored the differences between LGBTQ+ and heterosexual individuals in the levels of tested variables. A total of 414 participants took part in this study, of whom most study participants were young adults (M = 24.50; SD = 6.94). Of those, 130 participants identified themselves as heterosexual, while 284 declared themselves members of the LGBTQ+ community. The Satisfaction with Life Scale, Plopa’s Attachment Styles Questionnaire, and the State-Trait Anxiety Inventory were used. Data demonstrate that anxiety and attachment styles were significant predictors of life satisfaction in the LGBTQ+ community. Moreover, LGBTQ+ individuals had higher non-secure attachment styles and anxiety scores, and lower life satisfaction scores in comparison to heterosexual individuals. The presented study is the first Polish study to test such relationships in the Polish LGBTQ+ community. It draws attention to lower life satisfaction among study participants representing the LGBTQ+ community in comparison to cisgender heterosexual individuals. This indicates that researchers, clinical specialists and practitioners should try to improve the levels of subjective well-being in Polish LGBTQ+ individuals, for example, through psychoeducational and supportive programs. In addition, the presented results highlight the possible important role of attachment styles in the functioning of the presented group.

## 1. Introduction

The situation of the Polish LGBTQ+ community is very worrying. The current President of Poland, Andrzej Duda, publicly labelled the LGBTQ+ community an “ideology that is worse and more harmful than communism”; this issue has attracted the attention of the international media and has been discussed worldwide [1,2]. Discrimination by the head of state probably adversely affects the sense of security and well-being of LGBTQ+ people in Poland. Additionally, reports from independent research do not provide uplifting information. According to ILGA Europe (International Lesbian, Gay, Bisexual, Trans and Intersex Association), Poland had the lowest score on the Rainbow Europe report, an annual benchmarking tool created to monitor the legal and legislative situation of LGBTQ+ people in Europe [3]. This shows that Poland is one of the most discriminatory places in Europe. The political situation, legal policies, and society’s attitude towards people from the LGBTQ+ community in Poland clearly indicate the need to study the mental health and well-being of this group of individuals.

### 1.1. Minority Stress Theory

According to Meyer’s minority stress theory [4], LGBTQ+ individuals might perceive severe stress due to discrimination related to their gender identity or sexual orientation. This perceived discrimination and stress in the LGBTQ+ community might be the source of serious mental health outcomes, such as poor quality of life, lower life satisfaction, higher depression or severe anxiety [4,5]. According to Meyer’s theory, having a minority status can lead to additional stressors. These are related to individuals perceiving themselves as stigmatized and devalued. Having a minority identity is not only a source of stress, but an important modifier in the process of stress and coping with it. For this reason, Meyer [4] hypothesizes that LGBTQ+ individuals may more often struggle with mental problems due to excessive exposure to stress. Experiencing discrimination and stress related to sexuality can lead to a sense of alienation, lack of self-acceptance and a sense of rejection from the environment. This can lead to a range of mental problems such as anxiety, depression, and even suicide attempts. Meyer [4] rightly notes that over the years, the LGBTQ+ community has become more and more visible, which has made it more likely to become the object of violence and discrimination. This violence can also occur in young adults. Meyer [4] bases his concept on a number of empirical studies that suggest that LGBTQ+ youth may be more vulnerable to minority-based persecution. This stress can significantly shape their functioning in all spheres of life, due to fear for their own safety. It is important to note that Meyer [4] himself indicates the limitations of his own theory. He believes that the differences in mental health and functioning between LGBTQ+ individuals and cisgender heterosexual individuals are likely to be due to sample bias, leading to an overestimation of the epidemiology of mental disorders in minority groups. LGBTQ+ people who expressed their sexuality have gone through a unique process that can heighten introspection. This can lead to greater ease in disclosing mental health problems in comparison to cisgender heterosexual individuals [4].

### 1.2. Subjective Well-Being Model

A growing amount of empirical studies show that one’s discrimination due to their minority status might negatively affect their life satisfaction [6,7]. Life satisfaction is a term present in one of the most commonly referred to theories in positive psychology, the subjective well-being model. Subjective well-being is a very broad term that is not defined as one fixed construct. According to Diener [8], subjective well-being can be defined as a cognitive and emotional evaluation of an individual’s life experiences. The cognitive aspect of subjective well-being concerns judgments and beliefs about an individual’s experiences, while the emotional aspect reflects the reaction to the events taking place in one’s life. Subjective well-being consists of four components: life satisfaction (the subjective evaluation of one’s life), positive affect (the feeling of positive emotions, such as happiness, enthusiasm, pride, etc.), negative affect (the sense of fear, despair, guilt, nervousness, etc.), and domain satisfaction (such as health, family, self-esteem, work, finances, and others). According to Diener’s concept [8], in order to be characterized by high subjective well-being, one should enjoy high satisfaction with life, and experience a high number of positive emotions and a low number of negative emotions. He believes that subjective well-being plays an important role in health. It has been proven that life satisfaction is a significant part of functioning and health; this is also true for LGBTQ+ community members [4,6,7,9].

### 1.3. Attachment Styles Concepts

According to Bowlby’s attachment theory [10], attachment styles are cognitive, emotional, and behavioral patterns that form in the first years of life, based on relations with the caregiver or an important person (attachment figure), and which encompass an individual’s beliefs, expectations, and behaviors. The quality of relations with the caregiver in early childhood creates a foundation based on which strategies, principles, and expectations regarding the building of interpersonal relationships are formed. They play a significant role in interpersonal and close relationships throughout one’s whole adult life. Based on Bowlby’s theoretical framework [10], Ainsworth has distinguished three attachment styles: secure, anxious ambivalent and anxious avoidant [11]. Empirical evidence suggests that attachment styles are important predictors of life satisfaction and well-being [12,13,14]. It has been proven that the secure attachment style forms the basis for creating and maintaining satisfying interpersonal relations, and is linked with greater life satisfaction [15,16,17]. On the other hand, people who have developed an avoidant or anxious ambivalent style display lower levels of life satisfaction and well-being [18,19]. One cause of this may be an absence of trust in loved ones, as well as a high level of aversion to forming intimate relationships among those exhibiting an avoidant attachment style. These individuals are characterized by a high level of discomfort associated with closeness and intimacy in relationships, as well as the tendency to become overly dependent [18]. However, the anxious ambivalent style is associated with a high level of fear over the durability of relationships and conviction of a low level of support from the other person. Particularly in intimate relationships, people who have developed an anxious ambivalent attachment style tend to ‘stick’ to their partners, forcing their partners to continually pay attention to them and maintain an excessive closeness [20]. 

### 1.4. Aim of This Study

**Figure 1:**
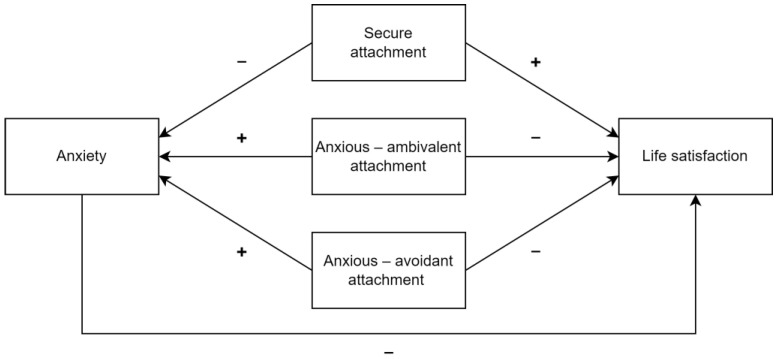
Visualization of the hypothesized relationships.

Research from different countries has confirmed that attachment styles, anxiety, and life satisfaction in the LGBTQ+ community are related to each other [21,22,23]. Unfortunately, none of the available studies have verified these relationships among Polish LGBTQ+ individuals. Therefore, this study aimed to verify the role of attachment styles in anxiety and life satisfaction in the Polish LGBTQ+ community. Based on Meyer’s minority stress theory [4], Bowlby’s attachment theory [10], the Ainsworth attachment framework [11] and Diener’s subjective well-being model [8], and provided empirical evidence, this study hypothesized that (1) secure attachment style will act as a positive predictor of life satisfaction in LGBTQ+ community in Poland, and a negative predictor of their anxiety levels; (2) anxious ambivalent and anxious avoidant attachment styles will act as negative predictors of life satisfaction in Polish LGBTQ+ individuals, and positive predictors of their anxiety levels; and (3) anxiety will act as negative predictor of life satisfaction scores in the studied sample. Based on the theoretical framework, both anxiety and life satisfaction will be treated as dependent variables, whereas anxiety and attachment styles will be treated as possible predictors. See Figure 1 for the visualization of the hypothesized model.

## 2. Materials and Methods

### 2.1. Participants and Procedure

Unfortunately, the presented study presents the results only based on cisgender LGBTQ+ individuals, because it failed to reach a sufficient number of gender minority representatives (such as transgender or non-binary individuals). Additionally, it was decided to recruit cisgender heterosexual individuals as a comparison group. It was decided to recruit a comparative group in order to show whether people from the LGBTQ+ community function at a similar level to heterosexual individuals. Verifying whether cisgender LGBTQ+ individuals function as well as cisgender heterosexual individuals may be crucial in the discussion of the results and directions of future research. A total of 414 cisgender participants took part in this study. All study participants were in a committed relationship, which was required for the attachment styles’ measurement (see the Measures Section 2.2). The studied group included 130 people self-identifying as heterosexual individuals and 284 participants who declared themselves part of the LGBTQ+ community (see Table 1 for more detailed information). Study participants were recruited through various Polish support groups and LGBTQ+ associations. To ensure the reliability of the received answers, the questionnaires were shared with the support groups and associations with a request that the invitations to the study should be sent by one of its members rather than one of the members of the research team. This decision was made in order to increase the sense of privacy and security of the LGBTQ+ individuals who took part in this study. It is important to note that the study participants were informed that the mentioned study was being conducted by the authors of this manuscript and that the proposed procedure was performed in order to make their answers fully anonymous. On the other hand, data from heterosexual participants were obtained from Polish charities and foundations whose work is not related to the LGBTQ+ community. The same procedure has been implemented during the recruitment of this group of study participants. When obtaining a comparison group, it was verified whether those participants also belong to the LGBTQ+ community. Based on the participants’ declarations, it can be stated that during the recruitment of heterosexual cisgender people, individuals from the LGBTQ+ community were not encountered.

The study participants were instructed to complete a short questionnaire through a specially prepared website designed to ensure full anonymity of the gathered data. They were informed that they could stop answering the questionnaires at any time. Informed consent was obtained from all subjects involved in the study. Incomplete questionnaires were not included in the analysis. The presented project adhered to the guidelines of the Bioethics Committee at the Institute of Medical Sciences of Opole University in Poland. According to the committee, the need for ethical approval was waived, since due to the cross-sectional and non-experimental nature of the presented project, it did not raise any ethical concerns (application number 2/KB/12/2019).

### 2.2. Measures

Attachment styles were measured using Plopa’s Attachment Styles Questionnaire (KSP) [24], which measures attachment styles in partner relationships. It was constructed based on Bowlby’s [10] and Ainsworth’s [11] theoretical frameworks. It is a 24-item scale containing 3 subscales of 8 items each, where each item is assessed on a 7-point scale. These questionnaire statements relate to the feelings that an adult person usually experiences when in a committed relationship. The subscales are (1) secure attachment, (2) anxious ambivalent attachment, and (3) anxious avoidant attachment. Higher subscale scores indicate the greater manifestation of the measured attachment styles in one’s behavior [24]. The reliability coefficients in the presented study were satisfactory (Cronbach’s alpha = 0.84; McDonald’s omega = 0.85).

Anxiety was operationalized using the State-Trait Anxiety Inventory (STAI), which measures anxiety as a relatively stable personality trait (X-2) and a temporary, situational state (X-1) [25]. Only the X-2 subscale was taken into the analysis. This subscale contains 20 items, to which study participants respond using a 4-point scale. Higher STAI scores indicate a higher anxiety level [25]. In the presented study, this scale shows very good internal consistency (Cronbach’s alpha = 0.83; McDonald’s omega = 0.84).

Life satisfaction was measured using the Diener’s Satisfaction With Life Scale (SWLS). SWLS is a five-item scale used to measure global life satisfaction. It consists of 5 items on a 7-point scale [26]. It was constructed based on Diener’s subjective well-being theory [8]. Higher SWLS scores indicate higher life satisfaction [26]. It had good internal consistency in the presented study (Cronbach’s alpha = 0.87; McDonald’s omega = 0.88).

### 2.3. Statistical Analysis

Differences between the studied groups were verified with a *t* test analysis. The relationships between the studied variables were measured with Pearson’s r correlation. Due to two possible dependent variables, the role of the tested predictors was verified with the path analysis approach. A level of α = 0.05 was adopted as the threshold value for statistical significance in all the analyses.

## 3. Results

First, we decided to analyze whether LGBTQ+ individuals differ from heterosexual individuals in their levels of the studied variables: attachment style, anxiety, and life satisfaction scores. Due to the insufficient number of asexual and bisexual individuals (see more detailed information in Table 1), it was decided to treat the LGBTQ+ community sample as a homogenous group. For his purpose, the *t*-test analysis for the independent sample was used. Additionally, Cohen’s d effect size measure was calculated in order to highlight the size of the verified between-groups differences. The *t*-test analysis results showed that LGBTQ+ individuals had significantly higher scores of anxious ambivalent attachment scores (moderate effect size), anxious avoidant attachment scores (small effect size), and anxiety (small effect size). On the other hand, heterosexual individuals had significantly higher life satisfaction (small effect size). There were no significant differences in the levels of secure attachment style. For detailed results, see Table 2.

Later, the relationship between tested variables in LGBTQ+ individuals was measured with the use of Pearson’s r correlation. Life satisfaction was positively related to the secure attachment scores (moderate effect size), and negatively to anxious ambivalent style (large effect size), anxious avoidant style (moderate effect size) and anxiety (large effect size). Additionally, the anxiety scores were negatively related to the secure attachment style scores (moderate effect size), and positively to the anxious ambivalent style (large effect size) and anxious avoidant style (moderate effect size). This means that individuals with higher scores of secure attachment style and lower scores of the anxious ambivalent and anxious avoidant styles and anxiety showed higher life satisfaction. Moreover, low levels of anxiety in the presented sample of LGBTQ+ individuals were associated with a greater intensity of secure attachment style and lower scores of anxious ambivalent and anxious avoidant styles. For more detailed results, see Table 3.

**Figure 2:**
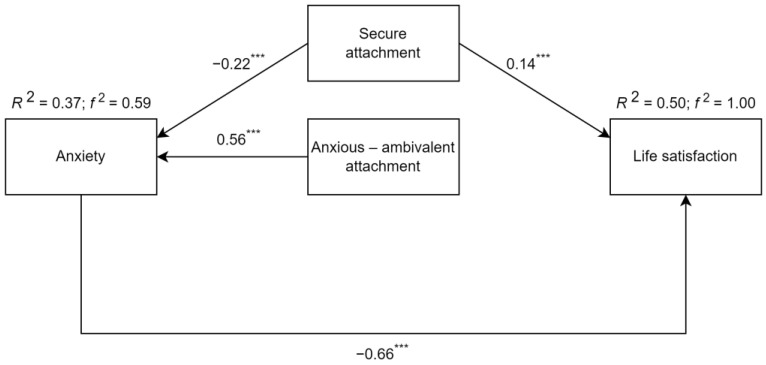
Standardized regression coefficients for the tested path analysis model. Note: *** *p* < 0.001.

Due to the limitations of the correlation analysis, we decided to analyze a path model based on the hypothesized relationships (for more information, see Figure 1). For this purpose, the path analysis with an ML (maximum likelihood) estimator was calculated. The initial model’s fit indices did not reach acceptable threshold values. Based on the proposed modification indices, the model was adjusted by deleting the insignificant paths. Figure 2 presents the values of standardized coefficients of the relationships between tested variables in the final model. Secure attachment was a significant predictor of both anxiety and life satisfaction in LGBTQ+ individuals. On the other hand, the anxiousambivalent attachment scores were significantly related only to anxiety results. The State-Trait Anxiety Inventory results were a significant predictor of the Satisfaction With Life Scale scores. The presented model’s fit coefficients were satisfactory (CMIN/df = 10.888; CLI = 0.947; SRMR = 0.101). Cohen’s f^2^ values indicate that the proposed model explained a large amount of the variance (for more detailed information, see Figure 2). Secure attachment and anxious-ambivalent attachment scores were responsible for the 37% of the anxiety score variance On the other hand, secure attachment style and anxiety scores were responsible for 50% of the life satisfaction variance. Based on the presented R^2^ values, it can be concluded that multicollinearity was not detected in the tested model (VIF coefficient values ranged between 1.59 and 2.00). Corresponding results were achieved using a lower-tier multiple regression analysis. Therefore, it was decided to report only the results of the path analysis.

The presented path analysis validated the results of Pearson’s r correlation. Based on the presented results of the correlation analysis and path model, it can be concluded that life satisfaction was significantly related only to two of its possible predictors: anxiety and secure attachment scores. On the other hand, secure attachment style was a negative predictor of anxiety, and anxious ambivalent style was its positive predictor. This excludes the possible significant role of anxious avoidant style scores in the levels of anxiety and life satisfaction, which was indicated in the correlation analysis results. It highlights that the other two attachment styles were more important in the case of the presented sample of Polish LGBTQ+ individuals’ selected measures of well-being and mental functioning.

The tested path model might have suggested the existence of possible indirect effects. Due to the cross-sectional nature of the presented data, those effects were not analyzed, since the mediation analysis is causal in its nature, and the presented study does not allow us to draw cause-and-effect-type relationships.

## 4. Discussion

The presented study was the first to analyze the relationship between attachment styles, anxiety, and life satisfaction in Polish LGBTQ+ individuals. Moreover, it has also compared the results of the tested LGBTQ+ community members with the results of cisgender heterosexual individuals. The results suggest that the tested LGBTQ+ individuals living in Poland had higher scores of anxious ambivalent and anxious avoidant attachment styles, as well as anxiety scores, and lower life satisfaction in comparison to heterosexual individuals. What is more, the analysis showed that anxiety and secure attachment style were the most important predictors of LGBTQ+ individuals’ life satisfaction among all tested variables.

The results indicated lower levels of life satisfaction among members of the Polish LGBTQ+ community. Similar conclusions have been drawn in many other studies, such as Powdthavee and Wooden’s [7], Perales’s [27] and Bejakovich’s [28]. Researchers perceive the sources of this lower satisfaction to result from different things, such as the worse socio-economic situation of LGBTQ+ individuals, but also their greater likelihood of remaining single, the absence of the possibility to formalize one’s relationship, and the smaller likelihood they will have children [7,27,28]. It is obvious that the stigmatization, intolerance, and homophobia frequently experienced by non-heterosexual individuals also probably plays a significant role in their levels of well-being [4,21,23,28,29]. This claim has a strong basis in Meyer’s [4] theory of minority stress presented in the introduction. Perhaps these phenomena not only reduced the levels of life satisfaction of the studied sample, but also increased their levels of anxiety, which were significantly higher among individuals in the LGBTQ+ group in comparison to cisgender heterosexual individuals. An important relationship between levels of anxiety and the experience of discriminatory behaviors has been demonstrated by Reitzel et al. [30]. Their studies indicated significantly higher levels of anxiety among LGBTQ+ individuals, particularly demonstrated by cognitive symptoms. Higher levels of anxiety among non-heterosexual groups were also confirmed in Swedish studies [31]. This phenomenon is observed irrespective of sex, and affects homosexual [32] and bisexual individuals [33]. Researchers perceive the causes of significantly higher levels of anxiety in the examined group in spheres analogous to those that reduce the feeling of life satisfaction. These significant predictors most often include the experience of discriminatory behavior [30], the absence of self-acceptance, or the absence of acceptance of one’s sexual orientation [34]. Those suggestions are also in line with Meyer’s theoretical model of minority stress [4].

Other variables significantly distinguishing the studied groups were attachment styles. Significant differences were present in both of its non-secure forms (anxious ambivalent and anxious avoidant), which were significantly higher in the group of LGBTQ+ individuals in comparison to cisgender heterosexual participants. These results are consistent with reports from a different study conducted among residents of Iran; its authors believe that these results are rooted in distinct developmental experiences and negative social attitudes towards LGBTQ+ people [35]. It is assumed that one’s attachment styles, formed in early childhood, remain relatively stable throughout life and impact one’s social and psychological functioning. People with a high level of anxious ambivalent style can have a negative view of self, low self-esteem, fear of rejection, and be emotionally dependent. A high level of avoidant style can be linked with negative opinions of others, a tendency to demonstrate mistrust towards people in our surroundings, and feelings of discomfort in situations of intimacy. People with a secure attachment style can manage negative emotions and seek support when they need it; this also facilitates the formation of a positive LGBTQ+ identity [10]. The mentioned results confirm the correctness of Bowlby’s [10] and Ainsworth’s [11] theoretical frameworks, and also the possibility of their application in the case of the functioning of LGBTQ+ individuals.

This study provides some interesting results, but it is not free of limitations. First, due to the cross-sectional nature of the study, it is impossible to draw any cause-and-effect-type conclusions. The presented hypothetical model may suggest a one-sided relationship. Those assumptions are based on the presented theoretical frameworks. In order to verify those assumptions, longitudinal and experimental studies should be carried out. Second, none of the sociodemographic variables other than gender identity, sexual orientation, and age were measured. As shown in the discussion, sociodemographic differences might play a significant role in the differentiation of tested variables. Even the presented theoretical framework (see Meyer’s minority stress theory description in the Introduction) suggests that variables such as financial situation may prove to be an important predictor of life satisfaction in minority groups. In addition, it is possible that different measures of psychological functioning might play a more important role in LGBTQ+ individuals’ life satisfaction than those that were measured in the presented study. For example, feelings of discrimination (e.g., heterosexism), self-esteem, or perceived social support can correlate with life satisfaction in minority groups. Therefore, future studies should also control those variables. The small amount of the tested variables in the presented study significantly limits the possibility of drawing conclusions. A wide range of measured constructions allows for their mutual verification, and shows which of them plays the most important role. Unfortunately, this study was limited to testing only two correlates of life satisfaction in Polish LGBTQ+ individuals: anxiety and attachment styles. This study did not examine single people and attachment styles in various spheres of life. This was due to the requirements of Plopa’s Attachment Styles Questionnaire [24], which measures attachment styles in romantic relationships in people in committed relationships. It is also important to explore the role of attachment styles in non-romantic relationships, such as friendships or household relations. Moreover, it is impossible to treat the proposed path analysis model as universal. The socio-demographic data presented in Table 1 do not allow us to compare the proposed path analysis model between groups. Those differences should be tested with a measurement invariance analysis, where the construct and metric equivalency are verified. Therefore, further research focused on Polish LGBTQ+ individuals should be carried out on a much larger sample of respondents, in order to be able to verify whether the proposed models are the same for all studied subgroups. Lastly, the presented study did not include any transgender, non-binary, agender, asexual, or other individuals, and the sample size of some LGBTQ+ subgroups was not representative enough. The study participants were recruited on the basis of targeted selection, and not from the general population. In order to ensure better representativeness of the presented results, the respondents should have been recruited in a more random way (e.g., via nationwide studies).

## 5. Conclusions

This manuscript presents data showing that anxiety and attachment styles are important predictors of life satisfaction in the tested sample of the Polish LGBTQ+ community. It draws attention to the lower life satisfaction among study participants representing the LGBTQ+ community in comparison to cisgender heterosexual individuals. This indicates that researchers, clinical specialists, and practitioners should try to improve the levels of subjective well-being in Polish LGBTQ+ individuals, for example, through psychoeducational and supportive programs. In addition, the presented results highlight the possible important role of attachment styles in the functioning of the presented group. This shows that the sphere of interpersonal relations and attachment should be taken into account when working on reducing anxiety and increasing life satisfaction in LGBTQ+ individuals. It is important to notice that previous Polish studies have shown that transgender individuals have lower life satisfaction and higher perceived stress in comparison to cisgender individuals [36]. Therefore, it is very important that future studies include gender minority samples in research on attachment styles in the Polish LGBTQ+ community. The presented results provide interesting and important data, but they need to be replicated on a more representative sample.

## Figures and Tables

**Table 1:** Detailed characteristics of the studied sample.

		*M*	*SD*
Age	All participants	24.50	6.94
	Cisgender men	24.77	7.35
	Cisgender women	24.23	6.50
	Homosexual individuals	23.98	6.47
	Heterosexual individuals	26.20	7.82
	Bisexual and pansexual individuals	23.00	6.16
	Asexual individuals	22.00	2.16
		*n*	%
Gender identity	Cisgender men	211	50.97%
	Cisgender women	203	49.03%
Sexual orientation	Homosexual individuals	215	51.93%
	Heterosexual individuals	130	31.40%
	Bisexual and pansexual individuals	65	15.70%
	Asexual individuals	4	0.97%

**Table 2:** Comparison of the LGBTQ+ and heterosexual individuals using a *t*-test analysis.

	LGBTQ+		Heterosexual		*t* (412)	*p*	*LLCI*	*ULCI*	*d*
	*M*	*SD*	*M*	*SD*					
Secure attachment	41.77	9.43	42.45	9.68	−0.67	0.500	−2.659	1.300	0.07
Anxious ambivalent attachment	34.75	12.38	28.45	12.07	4.84	<0.001	3.746	8.862	0.52
Anxious avoidant attachment	21.92	9.35	19.19	9.39	2.75	0.006	0.782	4.679	0.29
Anxiety	51.53	10.47	47.83	10.68	3.31	0.001	1.504	5.891	0.35
Life satisfaction	18.28	6.51	21.03	6.19	−4.05	<0.001	−4.083	−1.415	0.43

**Table 3:** Relationship between attachment styles, anxiety and life satisfaction in LGBTQ+ individuals, analyzed using Pearson’s r correlation.

	*M*	*SD*	1.	2.	3.	4.
Secure attachment	41.77	9.43	-			
Anxious ambivalent attachment	34.75	12.38	−0.25 ***	-		
Anxious avoidant attachment	21.92	9.35	−0.72 ***	0.51 ***	-	
Anxiety	51.53	10.47	−0.35 ***	0.60 ***	0.46 ***	-
Life satisfaction	18.28	6.51	0.37 ***	−0.50 ***	−0.42 ***	−0.71 ***

Note: *** *p* < 0.001.

## Data Availability

The data can be made available from the corresponding author upon reasonable request.
